# Enthesitis as a component of dactylitis in psoriatic juvenile idiopathic arthritis: histology of an established clinical entity

**DOI:** 10.1186/s12969-015-0003-2

**Published:** 2015-02-28

**Authors:** Katherine SL Tuttle, Sara O Vargas, Michael J Callahan, Donald S Bae, Peter A Nigrovic

**Affiliations:** Division of Immunology, Boston Children’s Hospital, Boston, MA USA; Department of Pathology, Boston Children’s Hospital, Boston, MA USA; Department of Radiology, Boston Children’s Hospital, Boston, MA USA; Department of Orthopedic Surgery, Boston Children’s Hospital, Boston, MA USA; Division of Rheumatology, Immunology, and Allergy, Brigham and Women’s Hospital, Smith 516B, One Jimmy Fund Way, Boston, MA 02115 USA

**Keywords:** Psoriatic arthritis, Juvenile idiopathic arthritis, Psoriatic juvenile idiopathic arthritis, Enthesitis, Enthesitis related arthritis, Dactylitis

## Abstract

**Context:**

Imaging of dactylitis in adult psoriatic arthritis suggests a pathophysiological role for enthesitis. However, histological definition of the dactylitic digit is unavailable.

**Objective:**

We evaluated the role of enthesitis in dactylitis associated with psoriatic juvenile idiopathic arthritis (psJIA) in a child who underwent detailed imaging and histologic evaluation.

**Design:**

Radiographs, ultrasound and high-resolution magnetic resonance imaging were employed to define the locus of disease in two dactylitic digits from a 14-year-old girl with psJIA. Biopsies were stained with hematoxylin and eosin, CD3, CD20, CD4, CD8 and CD117 and examined via light microscopy.

**Results:**

Radiologic features of dactylitis included enhanced signal at digital entheses without accompanying synovitis or tenosynovitis. Histologically, finger and toe tissue exhibited hypervascular tenosynovium with a fibromyxoid expansion of fibrous tissue. This was accompanied by sparse to moderate perivascular lymphocytic inflammation consisting predominantly of T cells, with occasional admixed B cells and mast cells. Neutrophils and plasma cells were absent. Fibrocartilage exhibited reactive features including increased extracellular myxoid matrix, binucleation, and focal necrosis, without cellular inflammation. Fibrinous synovitis and mild reactive synoviocyte hyperplasia were seen.

**Conclusions:**

Dactylitis in psJIA bears a radiographic resemblance to the condition in adult psoriatic arthritis. Histologic hallmarks include an expanded mast cell-populated extracellular myxoid matrix, cartilage degeneration, and a T cell-rich perivascular inflammatory infiltrate. These findings help to define enthesitis as a clinicopathologic entity.

**Electronic supplementary material:**

The online version of this article (doi:10.1186/s12969-015-0003-2) contains supplementary material, which is available to authorized users.

## Background

The classification of juvenile idiopathic arthritis (JIA) is a work in progress [1]. Among the categories of JIA recognized by the International League of Associations for Rheumatology (ILAR), psoriatic JIA (psJIA) presents a particular diagnostic challenge [2,3]. The criteria for diagnosis of psJIA include: 1) arthritis and psoriasis or 2) arthritis and at least two of the following: dactylitis, nail pitting/onycholysis, or psoriasis in a first-degree relative, in the absence of specified exclusions [1]. The appearance of definitive cutaneous psoriasis lags behind the onset of arthritis in approximately half of patients, at times after an interval of 10 years or more [4-6]. Furthermore, while psoriasis and psoriatic arthritis display striking enrichment within families, a family history of psoriasis is not especially discriminating, as it is absent in many patients, while relatively common in the general population [7,8]. Patients with psJIA can resemble those with other disease subtypes, including oligoarticular JIA, seronegative polyarticular JIA, and enthesitis-related arthritis (ERA), and they are commonly diagnosed initially with another JIA subtype [6,9-14].

A key clinical feature recognized to favor the diagnosis of psJIA is dactylitis, defined as sausage-like or fusiform swelling of a finger or toe that extends beyond the joint margins to encompass the entire digit [6]. Between 20-40% of patients with psJIA have dactylitis; this proportion is even greater in children who present before the age of 6 years [5,6]. Interestingly, the biology of dactylitis may bear on classification within JIA. While joint swelling and tenosynovitis may contribute to dactylitis, they are rarely sufficient to yield a dactylitic appearance, as evidenced by the fact that dactylitis is not a feature of adult rheumatoid arthritis where destructive tenosynovitis is common. Digits have a high density of entheses, defined as sites of bony attachment for tendons, ligaments, and joint capsules. These, entheses include the anchor points for the “pulleys” that prevent tendons from bowstringing with digital flexion [15]. In adults, MRI and ultrasound data implicate enthesitis in dactylitis [15-18]. However, in pediatric rheumatology, arthritis with enthesitis in the absence of frank psoriasis is usually grouped under the term enthesitis-related arthritis (ERA) [1]. This distinction implies that enthesitis is not a core feature of psoriatic arthritis in children. If dactylitis in children were shown to reflect enthesitis in a radiographic or histological manner, then this would highlight the challenge of drawing a clear demarcation between psJIA and ERA.

With these considerations in mind, we report a patient who developed psoriasis and arthritis with dactylitis at the age of 14 years. Given concomitant immunosuppression for juvenile dermatomyositis (JDMS) and a history of travel abroad, our patient underwent an unusually detailed radiological and histological investigation to exclude an infectious cause of her digital swelling. Biopsy tissues provided a unique opportunity to characterize the histologic features of dactylitis, and we sought to study these samples to provide the first characterization of the dactylitic digit and to identify evidence in favor of, or against, enthesitis as a contributor to dactylitis in psJIA.

## Methods

Informed consent for review of the medical record was obtained from the patient’s family, and assent was obtained from the patient. Radiologic imaging included plain radiographs of the affected digits and magnetic resonance imaging studies of the fifth toe using coronal short tau inversion recovery (STIR) and sagittal T2 with fat suppression sequences. Biopsy material from the two dactylitic digits as well as the scalp were fixed in formalin and embedded in paraffin. Histologic sections were stained with hematoxylin and eosin. Immunohistochemical staining with antibodies to CD20, CD3, CD4, CD5 (all from Ventana/Roche, Tucson, AZ), CD8 (Leica, Wetzlar, Germany), CD117 (Dako, Carpinteria, CA) was conducted. All slides were examined via light microscopy. A Boston Children’s Hospital Institutional Review Board waiver was obtained for this case report.

## Case presentation

### Clinical and radiological investigation

**Figure 1:**
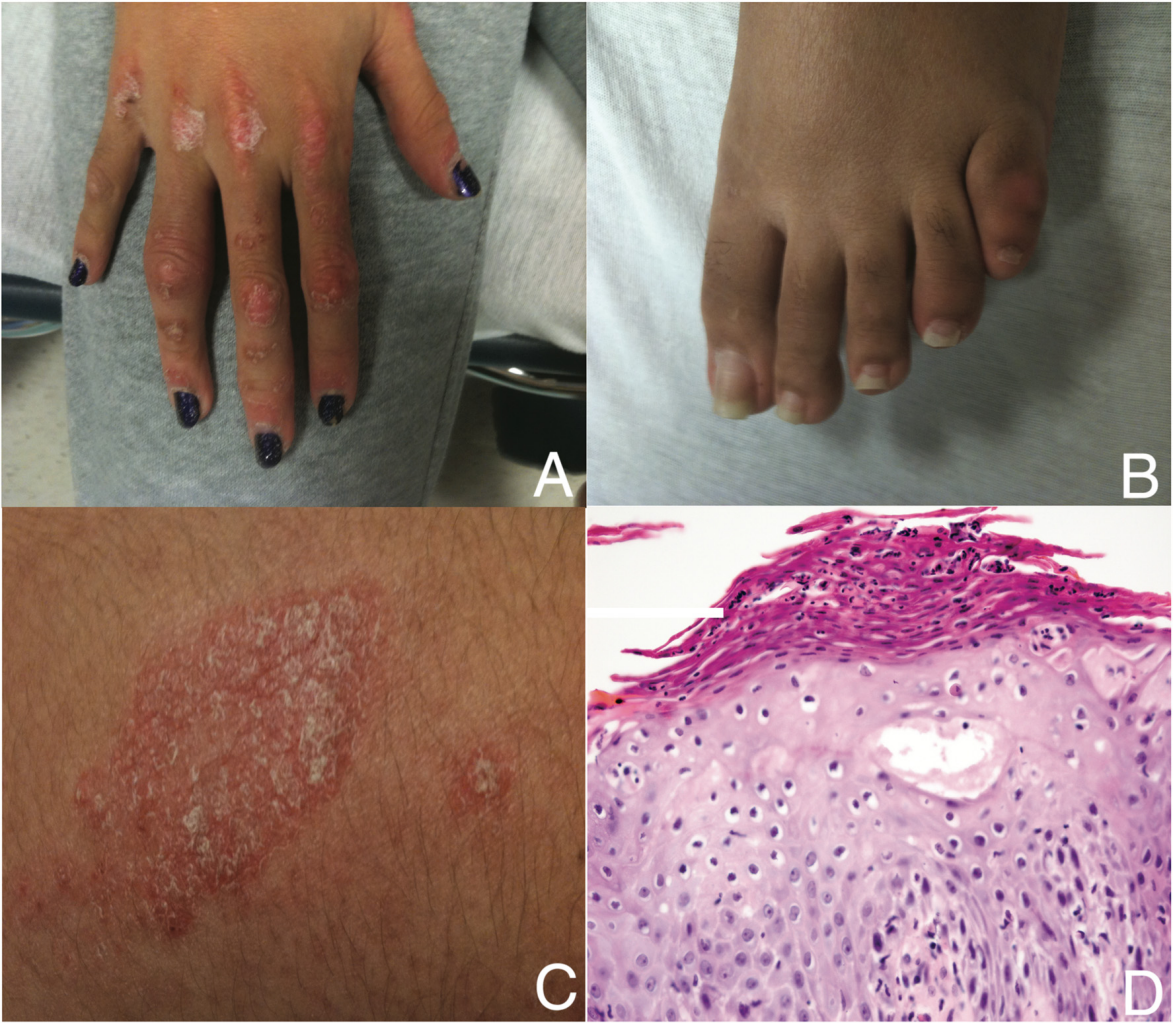
**Photographs depicting the clinical phenotype. A**. Dactylitis of the right fourth finger. **B**. Dactylitis of left fifth toe. **C**. Representative skin findings, here an annular plaque from right forearm characterized by erythema and overlying silvery scale, consistent with psoriasis. **D**. Scalp biopsy showing psoriasiform dermatitis, characterized by hyperkeratosis, parakeratosis, and neutrophils within the stratum corneum and spinous layer (hematoxylin and eosin, original magnification, 400×).

The patient was a female who was diagnosed with JDMS at age 3 years. The patient had been treated with corticosteroids, hydroxychloroquine, and methotrexate, but had persistent disease activity and developed multiple calcinoses and joint contractures, although without dactylitis or arthritis. At age 9 years, the patient moved from Central America to the United States to obtain further medical care. Her JDMS proved highly resistant to therapy, with new calcifications appearing even after therapy with pulsed corticosteroids, high-dose oral prednisone, methotrexate, cyclosporine, mycophenolate, and rituximab. Additional medications had included probenecid, diltiazem, colchicine, and atovaquone. At age 14, shortly after completing a course of cyclophosphamide and a trip to Central America, she presented with atraumatic fusiform swelling and tenderness of the right fourth finger and left fifth toe (Figure 1A and B). Skin examination revealed white, scaling, erythematous plaques on the knuckles (Figure 1A), frontal and occipital scalp, and bilateral dorsal forearms (Figure 1C). A scalp biopsy showed psoriasiform dermatitis (Figure 1D), supporting the clinical diagnosis of psoriasis.

**Figure 2:**
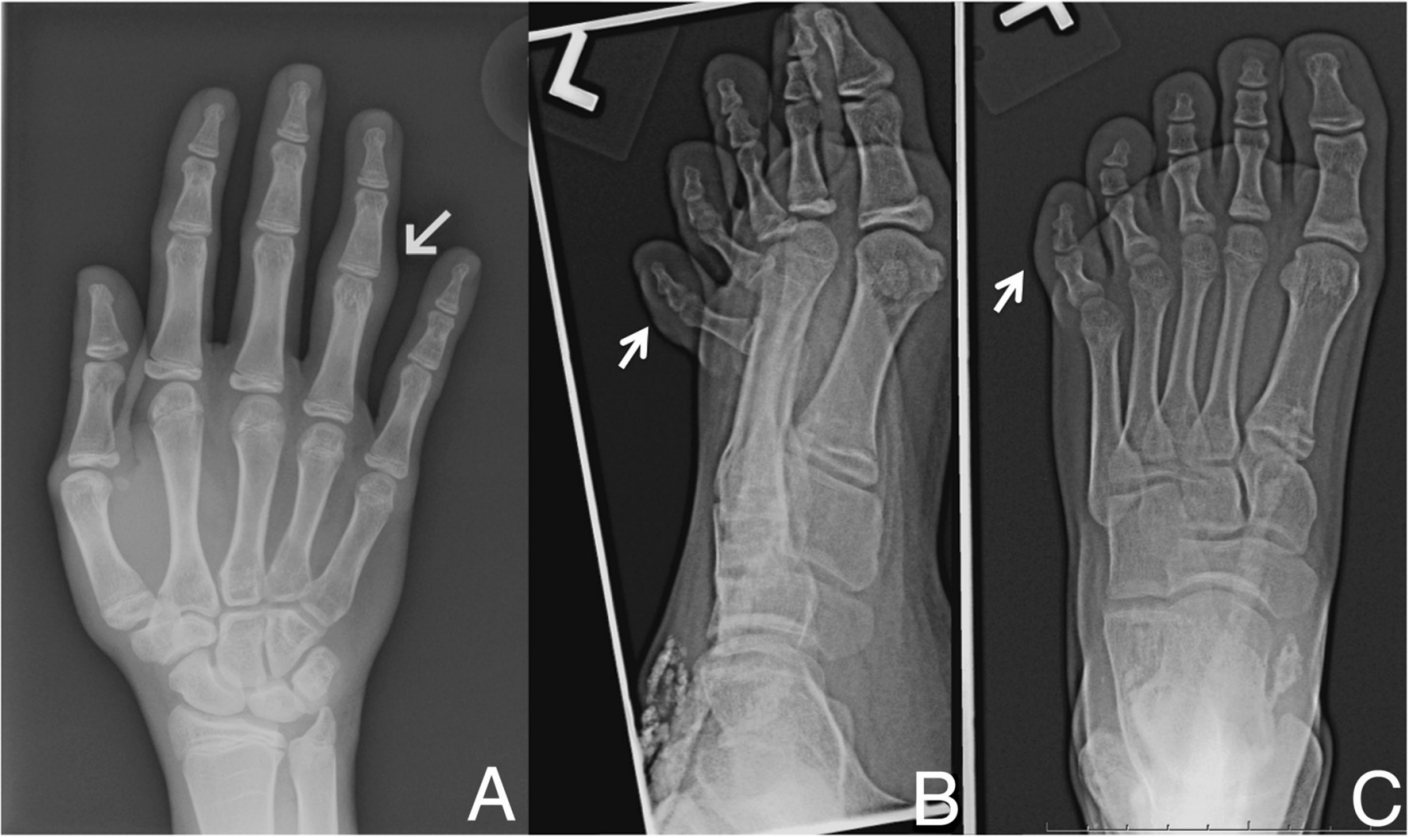
**Radiographic evaluation of dactylitis. A**. Radiograph of right hand (dorsal view) demonstrating diffuse swelling around PIP joint of right fourth digit, indicated by arrow. **B**. Radiograph of left fifth toe (oblique view) and **C**. Radiograph of the left fifth toe (dorsal view), both illustrating soft tissue swelling of left fifth toe, indicated by arrow. Subcutaneous calcifications related to the patient’s juvenile dermatomyositis are also evident.

**Figure 3:**
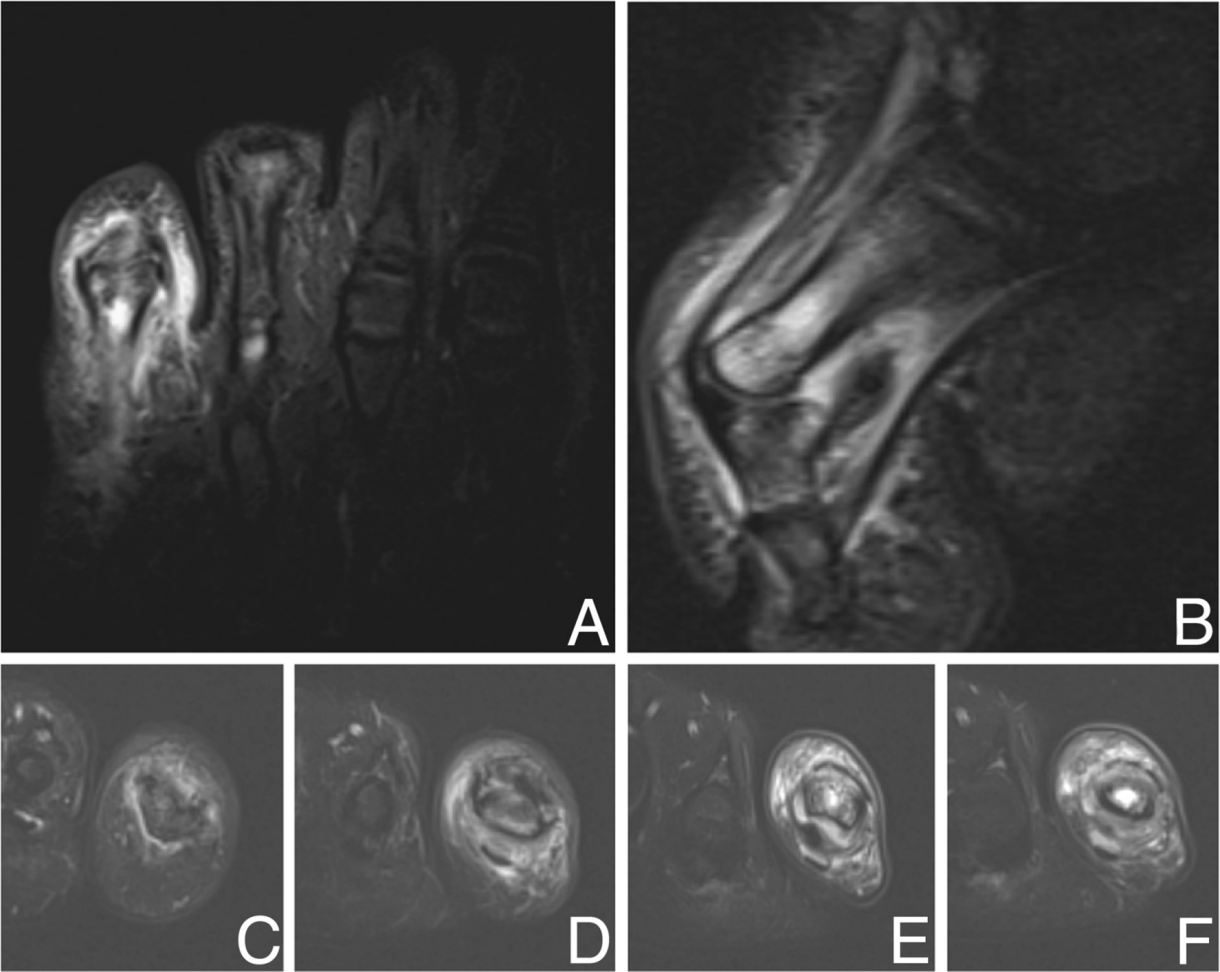
**Magnetic resonance imaging evaluation of dactylitis. A**. Coronal short tau inversion recovery (STIR), **B**. sagittal T2 with fat suppression, and **C**-**F**. axial T2 with fat suppression of the left fifth toe, proximal to distal sequences, demonstrating increased signal and gadolinium enhancement at the local entheses without associated tendonitis or synovitis.

Due to the concern over possible infectious dactylitis, including tuberculous [19], the patient underwent an unusually comprehensive evaluation. Chest radiograph, purified protein derivative test, sputum acid fast stain, and sputum mycobacterium culture were negative. Radiographs of the right hand and left foot showed soft tissue swelling of the right fourth digit and left fifth toe, without erosions or periosteal reaction (Figure 2A-C). Magnetic resonance imaging studies of the left fifth toe revealed swelling and contrast enhancement at the flexor tendon insertion with associated bone marrow edema, consistent with enthesitis, in the absence of either synovitis or tenosynovitis (Figure 3).

### Pathologic investigation

**Figure 4:**
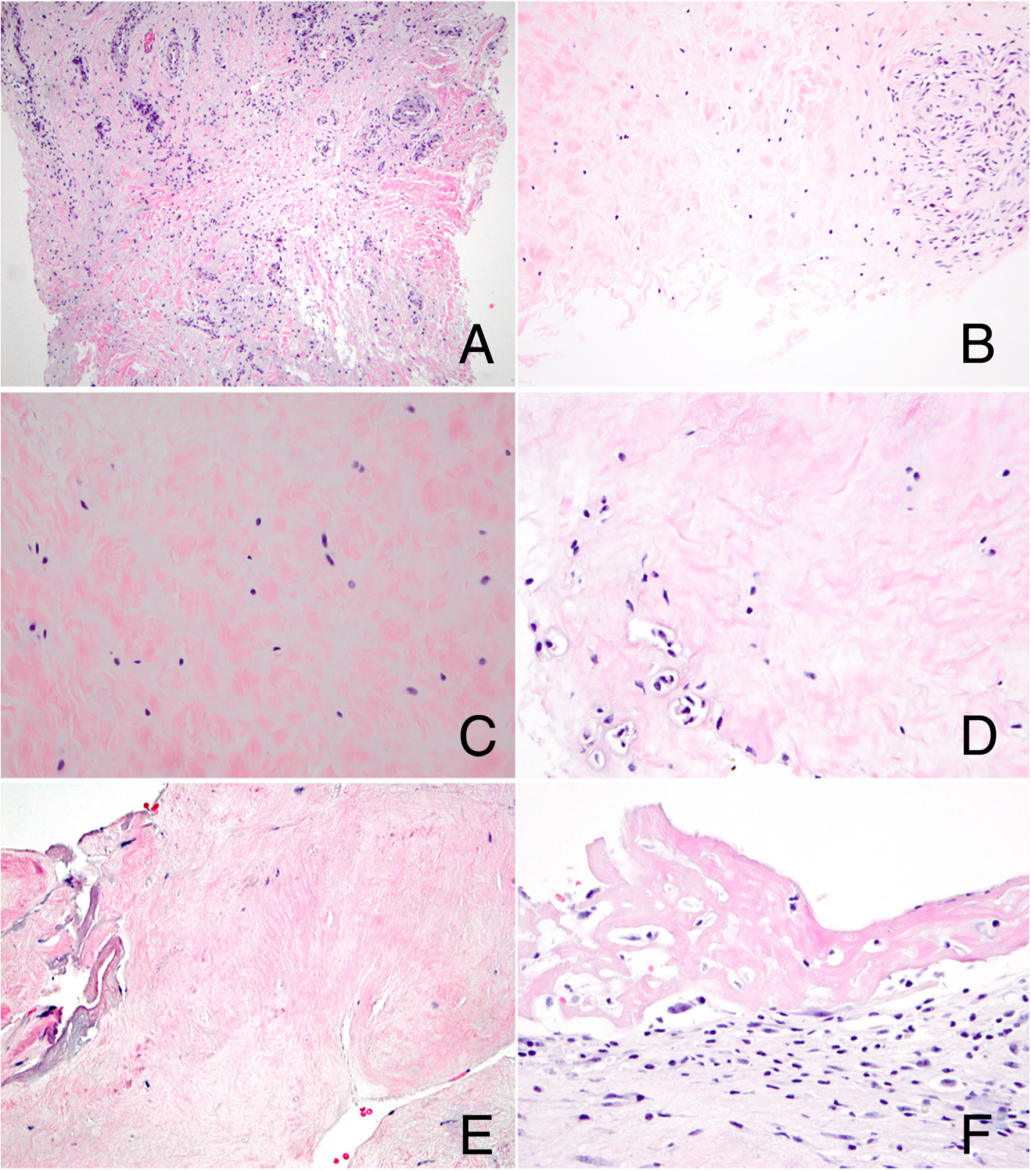
**Morphologic evaluation of dactylitis.** Both the affected finger and toe showed tenosynovial tissue with prominent vascularity and excess myxoid extracellular matrix (**A**, finger, original magnification, 100×); additionally, fibrocartilage showed a prominent myxoid component (**B**, toe, original magnification, 200×; **C**, toe, original magnification, 400×), areas of binucleation (**D**, toe, original magnification, 400×), and focal hypocellularity and coagulative necrosis (**E**, finger, original magnification, 400×). Fibrinous synovitis was also seen (**F**, finger, original magnification, 400×).

**Figure 5:**
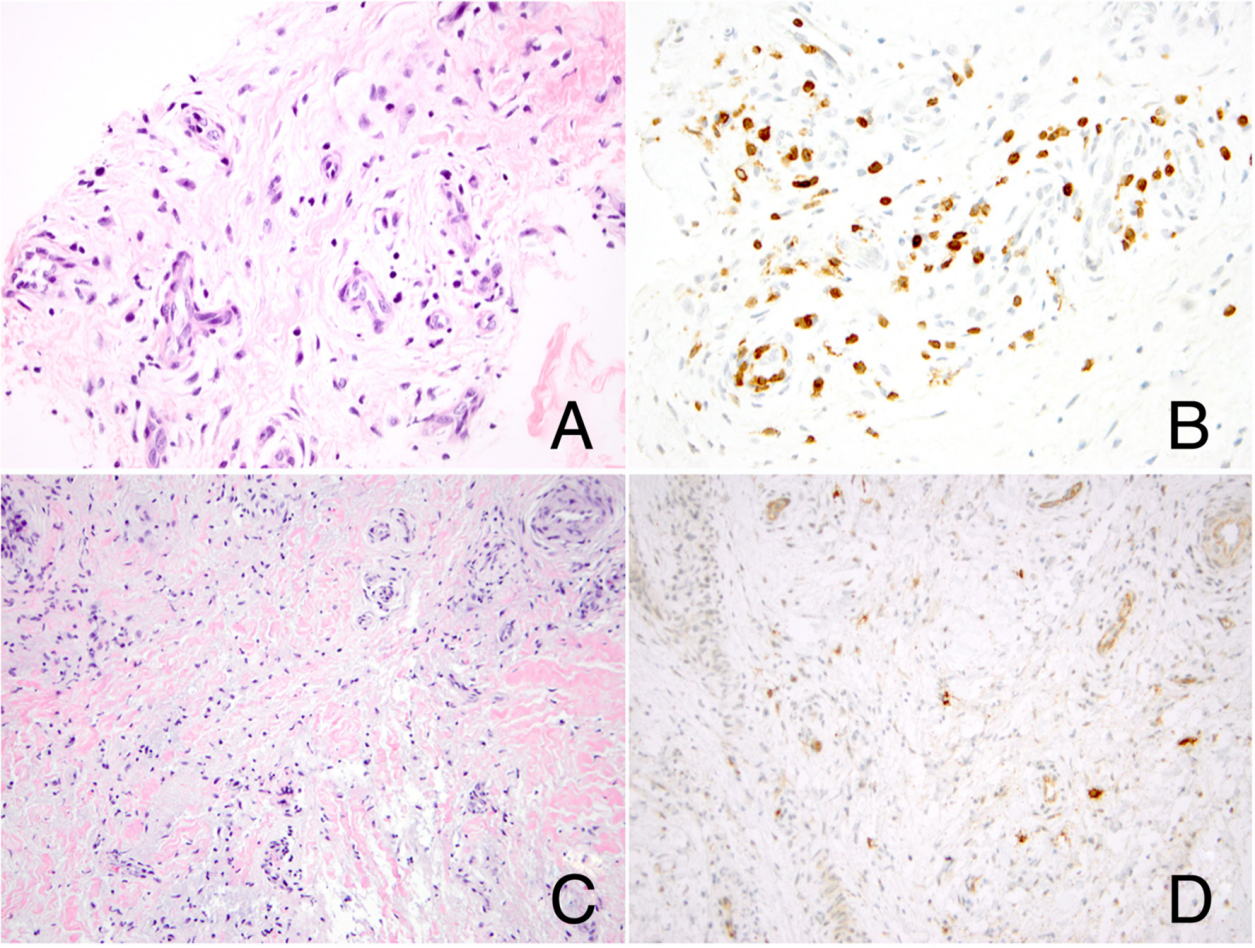
**Characterization of cellular inflammation.** Hypervascular tenosynovium showing perivascular inflammation consisting predominantly of T cells (finger; **A**, hematoxylin and eosin; **B**, CD3 immunostain; original magnification, 400×). In areas where myxoid matrix expands fibrovascular tissue containing densely collagenized tendon-like fibers, mast cells are regularly interspersed (finger; **C**, hematoxylin and eosin; **D**, CD117 immunostain; original magnification, 400×).

Clinical concern persisted regarding typical or atypical mycobacteria as a cause of the patient’s dactylitis, particularly in view of recent treatment with cyclophosphamide and planned tumor necrosis factor (TNF) blockade. Accordingly, deep soft tissue biopsies were performed on both swollen digits. Histologic examination of both the toe and finger showed hypervascular tenosynovium (Figure 4). Cellular inflammation (Figure 5) was mild in the toe and moderate in the finger, consisting predominantly of CD3-positive, CD5-positive, and CD4-positive T cells in both specimens, with rare CD8-positive cells. Sparse interspersed CD20-positive B cells and CD117-positive mast cells were also seen. The toe showed a small component of fibrocartilage that lacked cellular inflammation but showed reactive features including excess myxoid extracellular matrix and occasional binucleate and multinucleate chondrocytes. The finger revealed fibrinous exudate along a synovial surface and focal hypocellularity and coagulative necrosis of fibrocartilage. Neutrophils were not observed. No granulomas were seen, and acid-fast staining and mycobacterial culture of biopsy tissue were negative.

### Clinical course

The patient subsequently developed overt arthritis in several proximal and distal interphalangeal joints and in the left wrist, consistent with psJIA. She was treated with methotrexate and etanercept, resulting in clinical remission of dactylitis and partial improvement in her cutaneous psoriasis, although low-grade myositis and tumoral calcinosis persisted.

## Discussion

The extensive radiographic and histological evaluation prompted by our patient’s complex past medical history allowed us to examine the role of enthesitis in dactylitis-associated ILAR-defined psJIA. We found that enthesitis was prominent in the inflamed digits of our patient, and that this inflammation reflected both a chronic T cell-rich inflammatory infiltrate and stromal reaction, representing to our knowledge the first histologic definition of this clinical entity.

These findings have implications for the classification of childhood arthritides. They suggest a pathogenic continuity of psJIA with adult PsA, where enthesitis is recognized as a canonical clinical feature, and dactylitis is a highly specific physical finding [20]. Together with evidence of tender entheses on physical examination in some patients with psJIA [6], these observations blur the definitional boundary between psJIA and ERA, highlighting their pathophysiological similarities, and the potential utility of an overarching concept of spondyloarthropathy that encompasses both conditions.

Beyond these implications for disease classification, our histological findings advance the understanding of the clinicoradiographic entity known as enthesitis. “Enthesitis” as pathologic entity is not described in standard textbooks of surgical pathology [21] or bone pathology [22]. This may be in part due to the extreme rarity of enthesis biopsies and in part due to the lack of familiarity of pathologists with this lesion. Conceptual difficulty delineating the anatomic site may also contribute to the lack of pathologic recognition. The medical literature contains only scant reports of the histology of enthesitis, and to our knowledge no description of the histology of psoriatic dactylitis [23]. The lack of appreciation of enthesitis by pathologists is striking in contrast to the frequency of its use as a diagnostic term in the fields of radiology and rheumatology; this dichotomy underscores the need for a defined anatomic correlate.

The histology of enthesitis may provide insight into its pathobiology. Mast cells and myxoid expansion of the extracellular matrix have not been previously described in dactylitis, although they are encountered in the digital cellular proliferation known as superficial acral fibromyxoma, which like psoriatic arthritis affects predominantly the subungual/periungual region but is considered to be a benign neoplasm [24]. Mast cells are also often abundant in adult psoriatic synovium [25]. Their presence in enthesitis suggests that a component of the process is mediated via innate immunity, as has been increasingly suggested in the immune spondyloarthropathies [26,27]. The predominance of T cells in the lymphocytic infiltrate in the patient herein provides an interesting human correlate to recent studies demonstrating a key role for CD3+ lymphocytes in murine enthesitis [28]. Further study will be required to assess the relationship between the lymphocytes observed here and the IL-23 receptor-expressing, CD4/CD8-negative cells identified in mice; our tissue was unfortunately insufficient for these analyses [25].

Our patient was 14 years of age at time of diagnosis, and therefore falls into a psJIA phenotype that is clinically similar to adult psoriatic arthritis [6]. By contrast, younger patients with psJIA (age of onset less than 5 or 6 years) resemble in many ways children with early-onset oligoarticular and polyarticular JIA, including age of onset, female predominance and a tendency to be ANA positive, although they exhibit a distinct pattern of joint involvement and clinical enthesitis is less commonly observed [6,12]. Dactylitis is a particular hallmark of these younger patients, and it remains to be determined whether dactylitis in this younger subgroup is pathologically similar to dactylitis in older children, though clinical similarity suggests that this is probable.

The present report may also inform the discussion of the specificity of dactylitis for psJIA. 16-48% of adult patients with psoriatic arthritis have dactylitis, and this feature exhibits 95% specificity for psoriatic arthritis [20]. Under current pediatric classification criteria, dactylitis has been reported in a sizeable fraction of patients with either oligoarticular or polyarticular JIA [13]. This result raises two possibilities. Either dactylitis is substantially less specific in pediatric than in adult arthritis, or many pediatric patients with JIA and dactylitis have unrecognized psJIA. The current report suggests that the role of enthesitis in dactylitis extends to children as well as adults, supporting the latter possibility and favoring the hypothesis that psoriatic arthritis is distinct from other forms of JIA.

This study has several important limitations. We report a single patient, who was unusual in virtue of her history of JDMS and associated immunomodulatory treatment. It is possible that her psoriasis and psoriatic arthritis were triggered by treatment, though this appears unlikely because hydroxychloroquine exposure ended years before symptom onset and TNF inhibition was instituted only subsequently. Arthritis can complicate JDMS, raising concern that her condition should not properly be considered JIA. However, to the authors’ knowledge, dactylitis has never been reported in pediatric or adult dermatomyositis. Further, she had not experienced either arthritis or dactylitis from the time of JDMS diagnosis at age 3 through her presentation with dactylitis at age 14. Thus, an additional diagnosis of psJIA best explains this patient’s presentation with psoriasiform dermatitis and clinically typical psoriatic arthritis. Biopsies were taken at a single point in time, albeit within a few months of onset, and reflect only disease processes active at that time.

Given these caveats, it may be that the patient’s dactylitis reflects not just her psJIA but instead the sum of multiple immunological abnormalities. While we acknowledge this possibility, we doubt that it impedes the interpretability of our results. First, the patient’s clinical and radiographic findings were typical of those reported in adult PsA. Second, the patient exhibited a brisk response to usual psJIA therapy with TNF blockade, including resolution of dactylitis. While not diagnostic, this response is consistent with the expected effect of TNF inhibition on psoriatic dactylitis. Thus, while our findings need to be replicated in other patients, we nevertheless view this case to be highly informative as the first detailed description of the anatomy of dactylitis in psJIA.

## Conclusions

We describe the histologic correlate of clinically and radiographically bona fide digital enthesitis, in a child with psJIA. We find that enthesitis can be an important pathogenic contributor to psoriatic dactylitis in childhood. This result suggests that enthesitis should be regarded as a hallmark of psoriasis-associated arthritis across the age spectrum and illustrates the need to consider carefully the role of enthesitis in the classification of JIA.

## Consent

The patient and her family provided written consent for the publication of her case and its associated images in the medical literature.

## Abbreviations

ANA: Antinuclear antibody
ERA: Enthesitis related arthritis
JDMS: Juvenile dermatomyositis
JIA: Juvenile idiopathic arthritis
psJIA: Psoriatic JIA
PsA: (Adult) psoriatic arthritis
TNF: Tumor necrosis factor
